# Biofilm volume and acidification within initial biofilms formed in situ on buccally and palatally exposed bracket material

**DOI:** 10.1007/s00056-024-00515-4

**Published:** 2024-02-26

**Authors:** Micha Frederic Loewe, Katharina Doll-Nikutta, Meike Stiesch, Rainer Schwestka-Polly

**Affiliations:** 1 Department of Prosthetic Dentistry and Biomedical Materials Science, Hannover Medical School, Carl-Neuberg-Str. 1, 30625 Hannover, Germany; 2 Department of Orthodontics, Hannover Medical School, Carl-Neuberg-Str. 1, 30625 Hannover, Germany; 3 Lower Saxony Centre for Biomedical Engineering, Implant Research and Development (NIFE), Stadtfelddamm 34, 30625 Hannover, Germany

**Keywords:** Dental plaque, pH value, Orthodontic bracket materials, Confocal laser scanning microscopy, White spot lesions, Dentale Plaque, pH-Wert, Kieferorthopädische Bracketmaterialien, Konfokale Laserscanningmikrospkopie, White-Spot-Läsionen

## Abstract

**Purpose:**

Acidification by bacterial biofilms at the bracket/tooth interface is one of the most common problems in fixed orthodontic treatments, which can lead to white spot lesions (WSL) and caries. As lingual brackets were shown to exhibit reduced WSL formation clinically, the aim of this in situ study was to compare initial intraoral biofilm formation and acidification on bracket-like specimens placed buccally and palatally in the upper jaw as a possible cause for this observation.

**Methods:**

Intraoral biofilm was collected from splints equipped with buccally and palatally exposed test specimens, which were worn by 12 volunteers for a total of 48 h. The test specimens consisted of standard bracket material cylinders on top of a hydroxyapatite disc to represent the bracket/tooth interface. They were analyzed for three-dimensional biofilm volume and live/dead distribution by fluorescence staining and confocal laser scanning microscopy as well as for acidification by fluorescence-based pH ratiometry.

**Results:**

Similar general biofilm morphology with regard to volume and viability could be detected for buccally and palatally exposed specimens. For pH values, biofilms from both positions showed increased acidification at the bottom layer. Interestingly, the pH value at the top layers of the biofilms was slightly lower on palatally than on buccally exposed specimens, which may likely be due to anatomic conditions.

**Conclusion:**

Based on the results of this study, initial intraoral biofilm formation and acidification is almost similar on the bracket material/biomimetic tooth interface when placed buccally or palatally in the upper jaw. As lingual brackets were shown to exhibit reduced WSL formation clinically, future studies should investigate further factors like bracket geometry.

## Abstract (de)

**Zielsetzung:**

Die durch bakterielle Biofilme verursachte Ansäuerung an der Bracket/Zahn-Grenzfläche ist eines der häufigsten Probleme während der festsitzenden kieferorthopädischen Behandlung, das zu White-Spot-Läsionen (WSL) und Karies führen kann. Da linguale Multibracketapparaturen klinisch eine verminderte WSL-Bildung aufweisen, war das Ziel dieser In-situ-Studie, die initiale intraorale Biofilmbildung und Ansäuerung an bukkal und palatinal im Oberkiefer platzierten Bracket-ähnlichen Probekörpern als mögliche Ursache zu analysieren.

**Methoden:**

Mit Probekörpern bestückte Miniplastschienen dienten der intraoralen Biofilmgewinnung in 12 Probanden für insgesamt 48 h. Die Probekörper wurden bukkal und palatinal platziert und bestanden aus Standard-Bracketmaterialzylindern, welche auf Hydroxylapatitscheiben befestigt wurden, sodass die Bracket/Zahn-Grenzfläche imitiert werden konnte. Die Analyse des dreidimensionalen Biofilmvolumens und der Lebend/tot-Verteilung erfolgte durch Fluoreszenzfärbung und konfokale Laserscanningmikroskopie. Die Auswertung der Ansäuerung erfolgte durch fluoreszenzbasierte pH-Ratiometrie.

**Ergebnisse:**

In Bezug auf das Volumen und die Viabilität konnte für bukkal und palatinal exponierte Probekörper eine allgemein ähnliche Biofilmmorphologie nachgewiesen werden. An beiden Positionen war eine erhöhte Ansäuerung in den unteren Schichten des Biofilms zu detektieren. Interessanterweise war der pH-Wert in den obersten Schichten der Biofilme bei palatinal positionierten Probekörpern etwas niedriger als bei bukkal positionierten Probekörpern, was vermutlich durch anatomische Gegebenheiten bedingt ist.

**Schlussfolgerung:**

Basierend auf den Ergebnissen dieser Studie ist die initiale intraorale Biofilmbildung und Ansäuerung an der Grenzfläche zwischen Bracketmaterial und biomimetischem Zahn bei bukkaler bzw. palatinaler Platzierung im Oberkieferbereich ähnlich. Da linguale Multibracketapparaturen klinisch eine reduzierte WSL-Bildung aufweisen, sollten zukünftige Studien weitere Faktoren wie die Bracketgeometrie untersuchen.

## Introduction

Biofilms are structured microbial communities that are attached to a surface and surrounded by an extracellular matrix. In the mouth, they are responsible for many diseases, like caries and periodontal diseases, the two most prevalent medical threats in industrialized societies [10, 18, 43]. Dental caries is defined as a multifactorial, biofilm-mediated, diet-modulated, noncommunicable, dynamic disease resulting in mineral loss of dental hard tissues [21, 41, 48].

Biofilm formation begins shortly after the tooth surface has been cleaned. A pellicle composed of salivary proteins forms. This pellicle serves oral bacteria as attachment point to colonize the tooth surface [30, 39, 54]. When further bacteria colonize, the plaque grows into a three-dimensional structure surrounded by a matrix of self-produced extracellular polysaccharides [28]. In dental biofilms, the pH value of the extracellular matrix is the main virulence factor for the development of caries [57]. In order to gain energy, microorganisms in the plaque metabolize low-molecular-weight carbohydrates, thereby creating organic acids [10, 67]. This lowers the pH value, and minerals of the enamel such as calcium and phosphate can be dissolved. For tooth enamel, the critical pH value is 5.2–5.7 [28].

Enamel demineralization is also a significant risk factor and one of the greatest challenges during fixed orthodontic treatment [65]. The insertion of brackets, bands and arches complicates oral hygiene and promotes the accumulation of plaque [19].

The local acidification can subsequently lead to white spot lesions (WSL), which represent the first stage of caries formation [40]. WSL are milky white opacities of the enamel surface without cavity formation [59], which can already occur one month after the start of orthodontic treatment [46, 47]. The incidence of WSL is given in the literature up to 72.9% [53]. A 2015 meta-analysis concluded that the incidence of WSL during orthodontic treatment was 45.8% and the prevalence was 68.4% [59].

A significant reduction in the occurrence of WSL during orthodontic treatment was observed with a completely customized lingual multibracket appliance. The global incidence of new WSL for this treatment was determined to be only 3.19% for the entire dentition (teeth 17–47) [69]. When focusing on the upper incisors, it was shown that the subject-related incidence was 9.59% and the teeth-related incidence was 4.1% [36]. A comparison with previous studies on WSL incidence following conventional buccal appliances indicated a reduction by a factor 6.35 for the subject-related and a reduction by a factor 14 for the teeth-related WSL incidence [36]. Another study comparing WSL between lingual and buccal multibracket appliances found that the number of newly developing or progressive WSL was 4.8 times lower lingually than buccally [66]. In addition, the integrated fluorescence loss, measured using the quantitative light-induced fluorescence, was 10.6 times lower for lingual than for buccal surfaces [66].

According to these results, one could hypothesize that there is less biofilm formation on lingual than on buccal orthodontic brackets. As a consequence, acidification would be lower and fewer WSLs would occur. However, studies comparing biofilm formation and acidification lingually/palatally and buccally at the bracket–tooth interface have not yet been carried out.

Therefore, the objective of this in situ study was the three-dimensional investigation of initial intraoral biofilm formation and pH values on buccal and palatal bracket material. For this purpose, splints equipped with test specimens were exposed to the oral cavity of 12 volunteers for a total of 48 h. To quantify biofilm volume and live/dead distribution, the specimens were fluorescently stained and examined by confocal laser scanning microscopy (CLSM). The pH value within the biofilms was measured using pH ratiometry by the pH-sensitive ratiometric dye seminaphthorhodafluor-4F 5‑(and -6)-carboxylic acid (C-SNARF-4). Depending on the protonation, the dye shows a shift in its fluorescence emission detected by CLSM and can, thus, measure the extracellular pH value in the range from 4.5 to 7.0.

By combining the results of biofilm volume and pH value quantification, biofilm formation and acidification on buccally and palatally exposed bracket materials in the upper jaw were compared.

## Materials and methods

### Subject selection

The present study was approved by the ethical committee of the Hannover Medical School (amendment to ethic vote no. 8570_BO_S2019). Twelve individuals (6 women and 6 men) aged between 23 and 36 years (mean 27.6 years) participated in this study. Their healthy periodontal condition was assured by an initial periodontal screening including the probing depths (PD), the modified sulcus bleeding index according to Lange (SBI) and the modified approximal plaque index according to Lange (API) [28].

Exclusion criteria were general diseases, antibiotic treatment 6 weeks before participation, smoking, removable dentures, and pregnancy. All participants were informed about the objectives and interventional processes of the study and signed a consent form. The collected data were anonymized.

### Test specimen preparation

**Fig. 1:**
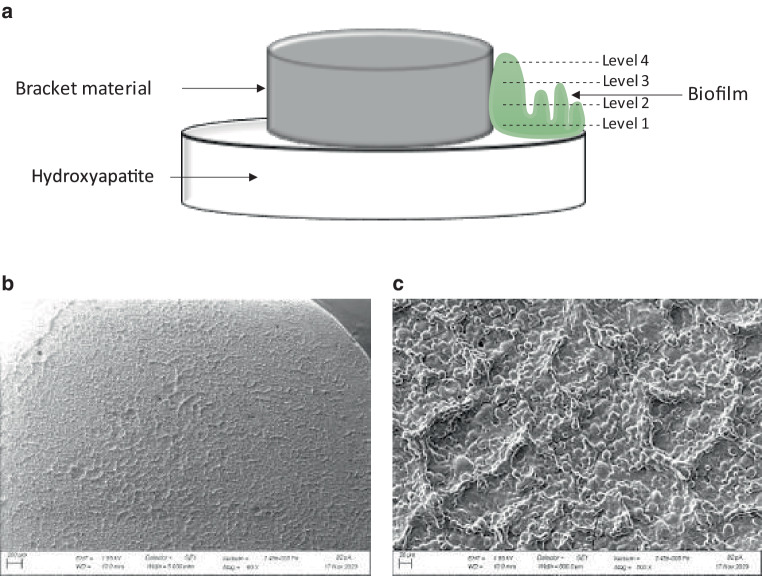
**a** Schematic representation of the test specimen setup with the investigated pH levels at the biofilm–hydroxyapatite bracket material interface. **b** Scanning electron microscopy images of the hydroxyapatite disc at different magnifications: scale bars corresponding to **b** 200 µm, **c** 20 µm **a** Schematische Darstellung des Probekörperaufbaues mit Kenntlichmachung der untersuchten Ebenen zur Bestimmung des pH-Wertes an der Biofilm-Hydroxylapatit-Bracketmaterial-Grenzfläche. **b** Rasterelektronenmikroskopische Aufnahme des Hydroxylapatit-Probekörpers bei unterschiedlichen Vergrößerungen: „scale bars“ entsprechend **b** 200 µm, **c** 20 µm

**Fig. 2:**
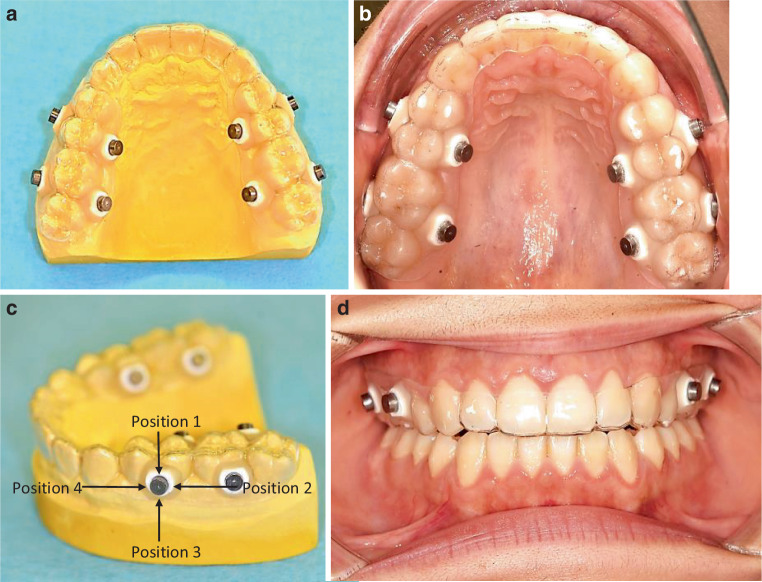
**a** Occlusal splint design: Occlusal splints with fixed specimens in the molar and premolar region on both the buccal and palatal sides of the first and second quadrants on a plaster model and **c** examined positions of the fixed specimen consisting of a hydroxyapatite specimen with bonded bracket-material. **b**, **d** Intraoral photos of the integrated occlusal splint (**b** occlusal view, **d** front view) **a** Konstruktion der Okklusionsschiene: Okklusionsschienen mit im Prämolaren- und Molarenbereich sowohl palatinal als auch vestibulär in den ersten und zweiten Quadranten befestigten Probekörpern auf einem Gipsmodell und **c** untersuchte Positionen der befestigten Probekörper, bestehend aus einem mit Bracketmaterial bestückten Hydroxylapatit-Probekörper. **b**, **d** Intraorale Ansichten der eingesetzten Okklusionsschiene (**b** okklusale Ansicht, **d** frontale Ansicht)

To mimic the bracket/teeth boundary, the test specimens consisted of standard bracket material cylinders on top of a hydroxyapatite disc (Figs. 1a and 2). The cylinders were provided by FORESTADENT® -Bernhard Förster GmbH (Pforzheim, Germany) and had a diameter of 3.5 mm and a height of 1.7 mm.

The hydroxyapatite discs were custom-made from hydroxyapatite powder with a particle size of 10 µm (Sigma–Aldrich, St. Louis, MO USA). The powder was processed to a mash with modelling liquid (VITA Zahnfabrik H. Rauter GmbH & Co. KG, Bad Säckingen, Germany) and filled into a silicon template of the required disc shape. Using a vacuum pump, the liquid was withdrawn through an ash-free filter (Rotilabo® round filter, Typ 13A, Carl Roth GmbH & Co. KG, Karlsruhe, Germany). The resulting filter cake was then sintered in a sintering furnace (sintering process: in 15 min up to 95 °C, maintain this temperature for 1 h, in 3 h up to 1500 °C, maintain this temperature for 2 h, in 2 h down to 1000 °C, maintain this temperature for 1 h and in 5 h down to 40 °C). The resulting hydroxyapatite discs had a diameter of 6 mm and a height of 1.5 mm. To test for basic biocompatibility, normal adhesion and growth of human fibroblasts on the discs was verified microscopically in preliminary experiments. The bracket cylinders were bonded directly on the hydroxyapatite discs with light cure adhesive paste (Transbond™ XT, 3M Unitek, Neuss, Germany), without prior etching with phosphoric acid to avoid impact of the hydroxyapatite surface quality.

### Splint design and in situ examination period

An upper jaw impression of every participant was taken with alginate (Alginoplast®, Kulzer GmbH, Hanau, Germany) and a plaster model was subsequently produced (SHERAALPIN gelb, Hartgips Typ 3, DIN EN 6873, Shera Werkstoff-Technologie GmbH & Co. KG, Lemförde, Germany). A thermoplastic deep drawing procedure (Erkodur, Erkodent® Erich Kopp GmbH, Pfalzgrafenweiler, Germany) was used to manufacture the occlusal splints.

The test specimens were placed buccally and palatally in the premolar and molar region in the first and second quadrants with flowable composite (Tetric EvoFlow A2, Ivoclar Vivadent AG, Schaan, Lichtenstein) on the occlusal splints (Fig. 2). In order to improve the adhesive bond, the desired areas were sandblasted with aluminum oxide (150 µm) beforehand (Sheraaluminiumoxid, SHERA Werkstoff-Technologie GmbH & Co. KG, Lemförde, Germany).

The specimen-equipped splints were worn in situ for 48 h by the participants. During this time, oral hygiene was suspended. The splints could only be removed for eating (stored in humid environment), but not longer than 40 min. After the examination period, the specimens were carefully removed from the splint without damaging the integrity of the biofilm and placed in phosphate-buffered saline (PBS, Sigma–Aldrich). The specimens were assigned to either a pH group or a live/dead staining group, based on predefined block randomization to exclude effects of the oral quadrant or the specific location.

### Live/dead fluorescence staining and biofilm volume quantification

In situ grown biofilms were fluorescent labeled using the LIVE/DEAD^TM^ *Bac*Light™ Bacterial Viability Kit (Thermo Fisher Scientific, Braunschweig, Germany). The fluorescent dyes Syto9® and propidium iodide were simultaneously applied as 1:4000 dilution in PBS according to the manufacturer’s recommendations. Specimens were fixed with 2.5% glutaraldehyde (Carl Roth GmbH + Co. KG) and placed in PBS for microscopy. Using the TCS SP8 confocal laser-scanning microscope (CLSM, Leica Microsystems GmbH, Wetzlar, Germany) three-dimensional images with a 400× magnification and a z-step-size of 5 µm were taken at four defined positions per specimen (Fig. 2c). The laser lines 488 nm and 552 nm as well as emission spectra of 500–550 nm and 650–750 nm were used to detect Syto9 and propidium iodide, respectively. The Imaris software package (Imaris 8.4, Bitplane AG, Zurich, Switzerland) was used to quantify biofilm volume and live/dead distribution.

### Microscopic biofilm pH analysis

Analysis of the biofilm pH was based on the protocol by Schlafer and Dige [57] using a pH-sensitive ratiometric dye. The biofilm covered specimens were immersed in 50 mM HEPES buffer pH 7.0 (Thermo Fisher Scientific) containing 7.5 µM SNARF®-4F 5‑(and-6)-carboxylic acid (Thermo Fisher Scientific) and 0.4% glucose (Carl Roth GmbH & Co. KG) and incubated for 45 min in 5% CO_2_ at 37 °C. Subsequently, fluorescence was recorded by CLSM (TCS SP8, Leica Microsystems GmbH) using a 488 nm laser line and detecting emission at 485–490 nm (surface reflection), 576–608 nm (green proportion), and 629–661 nm (red proportion). Three-dimensional images with 20 steps were taken at four defined positions on the specimens (Fig. 2c) at a magnification of 400×. To quantify the green/red ratio in different biofilm layers, tagged image file format (TIFF) files of four levels per position were exported using the Leica LAS X Core software (Leica Microsystems GmbH). Level 1 was defined as bottom layer with maximum surface reflection and level 4 as top layer of the biofilm. Levels 2 and 3 were evenly distributed in between (Fig. 1a). The software daime (digital image analysis in microbial ecology; University of Vienna, Vienna, Austria) [13] was used to delete bacterial and cellular biomass from the images. For every level, the green/red ratio of the remaining biofilm matrix was calculated with the ImageJ software (ImageJ 1.53e, Wayne Rasband, National Institutes of Health, Bethesda, MD, USA, http://imagej.nih.gov/ij/) according to the protocol by Schlafer and Dige [57].

To link green/red ratios to pH values a calibration experiment was performed. SNARF® (7.5 µM) was added to HEPES buffer with pH values ranging from 4.0 to 8.0 in steps of 0.2. Five images were taken per pH value by CLSM and processes as described above. Calibration was done using the GraphPad Prism software 8.4 (GraphPad Software, Inc., La Jolla, CA, USA) by interpolating a sigmoidal, 4PL, standard curve with logarithmic x‑axis.

### Statistical analysis

Sensitivity analysis of the experimental design was done using G*Power software 3.1.9.7 [20] for one-way analysis of variance (AVOVA) fixed effects, with the given total sample size of 48 (respectively 44) distributed in two groups, α = 0.05 and a statistical power of 0.8. Statistical comparison of biofilm morphology and pH was carried out with the GraphPad Prism software 8.4. Position effect of specimens on each splint did not have to be taken into account due to block randomization. Position effect within each specimen (Fig. 2c) was assessed by two-way ANOVA (biofilm volume and live/dead distribution) or by restricted maximum limitation (REML) mixed-effects model (pH) with Bonferroni’s multiple comparison correction. As the effect could be excluded, values for all positions on one specimen were averaged and used for further analysis. D’Agostino & Pearson omnibus test was performed to assess normal distribution of biofilm volume and live/dead distribution. Subsequently, Wilcoxon matched-pairs signed rank test was used to test for significant differences between palatal and buccal samples. To test for significant differences in pH values between palatal and buccal samples as well as between the different levels within one sample, REML mixed-effects model with Bonferroni’s multiple comparison correction was used. For all analyses, significance level was set to α = 0.05.

## Results

To analyze bacterial biofilm pH at the orthodontic bracket/tooth interface, test specimens consisting of cylindric bracket material on a hydroxyapatite disc were exposed in the upper jaw of 12 participants. To verify healthy oral conditions, initial periodontal screening was performed and revealed the following: PD of 1.5 ± 0.12 mm, SBI of 1 ± 0.02%, and API of 10.64 ± 0.03%. After 48 h of in situ biofilm growth, test specimen of 11/12 subjects (one subject withdrew from the study due to an acute temporomandibular disorder [TMD]) could be included in the study and were stained and analyzed by CLSM. With this experimental design, effects with an effect size >0.4 could be detected ensuring a statistical power of 0.8.

**Fig. 3:**
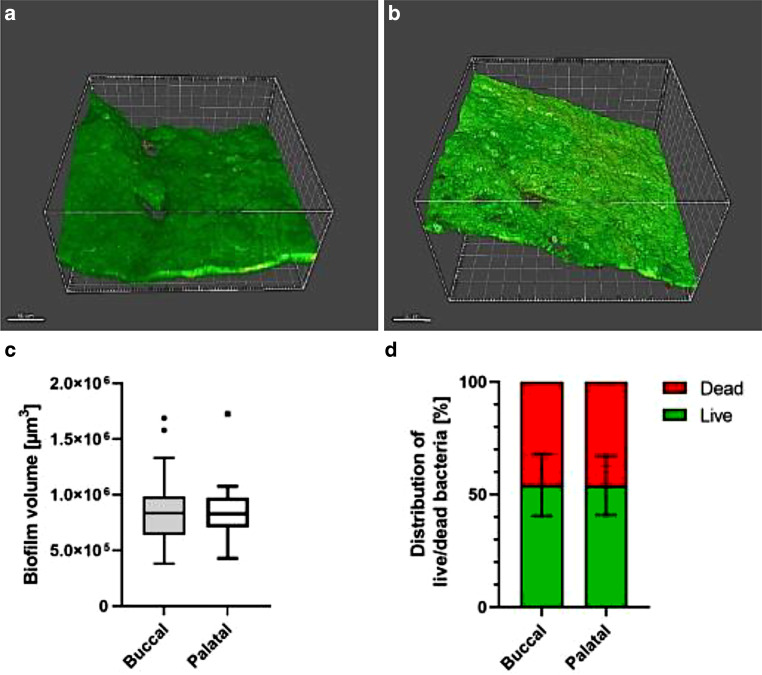
**a** Three-dimensional (3D) image reconstructions of confocal laser scanning microscopy (CLSM) data; magnification 400×. Scale bars: 50 µm. **a** buccal **b** palatal. Quantification of biofilm formation. Box-plot diagram of **c** biofilm volume and **d** live/dead distribution **a** Dreidimensionale Bildrekonstruktionen der durch CLSM (konfokale Laserscanningmikroskopie) gewonnenen Daten, Vergr. 400:1. „Scale bars“: 50 µm, **a** vestibulär **b** palatinal. Quantifizierung der Biofilmbildung **c** Box-Plot-Diagramm des Biofilmvolumens und **d** der Lebend/tot-Verteilung

Half of the specimens were analyzed for biofilm volume and live/dead distribution at the bracket/hydroxyapatite disc interface. Representative reconstructions of the biofilms are shown in Fig. 3a, b. They exhibited three-dimensional morphology and consisted of bacterial as well as human cells. As a specific position effect (Fig. 2c) could be excluded by statistical analysis, average values for buccally and palatally exposed specimens were compared. The average buccal biofilm volume was 8.79 × 10^5^ ± 3.31 × 10^5^ μm^3^ and the average palatal biofilm volume was 8.32 × 10^5^ ± 2.73 × 10^5^ μm^3^ (Fig. 3c). No statistically significant difference could be detected. The live/dead distribution was also almost similar between buccally and palatally exposed specimens with approximately 54% living and 45% dead cells (Fig. 3d).

**Fig. 4:**
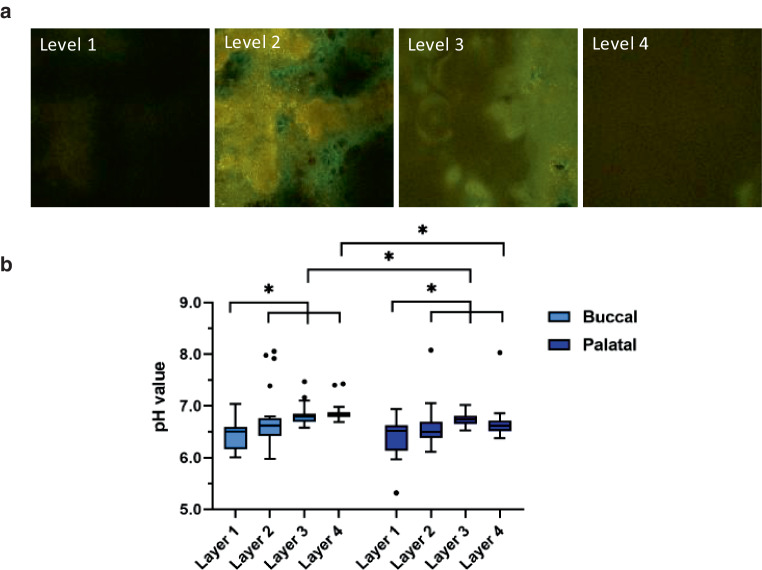
**a** Biofilm pH imaging of confocal laser scanning microscopy (CLSM); magnification 400×. **b** Box-plot diagram of pH values in different layers of the biofilms **a** pH-Wert Darstellungen innerhalb des Biofilmes mittels CLSM (konfokale Laserscanningmikroskopie), Vergr. 400:1. **b** Box-Plot-Diagramm der pH-Werte in den verschiedenen Ebenen der Biofilme

The other specimens were analyzed for pH values at different biofilm layers (Fig. 1a). For each position on the specimen (Fig. 2c), three-dimensional images of the biofilm were taken. From these images, four levels were selected for pH analysis (Fig. 4a). Level 1 was defined as bottom layer and level 4 as top layer of the biofilm. Level 2 and 3 were evenly distributed in between. As for biofilm volume a position specific effect (Fig. 2c) of the pH value could be excluded by statistical analysis, average values per level were calculated (Fig. 4b).

For buccally exposed specimens, the pH value steadily increased from pH 6.46 at the bottom layer (level 1) to pH 6.89 at the top layer (level 4). In contrast, for palatally exposed specimens, the pH was 6.40 at the bottom layer, increased up to pH 6.74 at level 3 and slightly reduced to pH 6.68 at level 4.

For biofilms grown on both oral positions, the pH value at the bottom layer was significantly lower compared to all other layers. When comparing both bottom layers (1 and 2) between buccal and palatal specimens, no differences could be detected. In contrast, the pH values of the top layers (3 and 4) were significantly lower palatally than buccally.

## Discussion

Common complications of orthodontic treatment are local demineralizations of the enamel that could lead to the formation of white spot lesions (WSL) and caries. They are caused by acid production of increased amounts of dental plaque around the brackets, due to inadequate oral hygiene during orthodontic treatment [7, 8, 59]. Interestingly, it has been shown that biofilm growth depends on intraoral location [5, 58]. In line with this, WSL showed a decreased clinical prevalence for lingual brackets [66, 69]. Therefore, the hypothesis of this study was that there is a difference in initial intraoral biofilm formation on buccally and palatally bonded brackets, resulting in different acidification. To address this hypothesis, this study quantified volume, live/dead distribution and pH value of initial biofilms formed on buccally and palatally exposed test specimens in the upper jaw of volunteers.

Prior to the study, healthy periodontal conditions of each participant were assured by an initial periodontal screening including probing depths (PD), modified sulcus bleeding index (SBI), and modified approximal plaque index (API) [28]. All values were within the normal range. Further risk factors influencing oral microflora, such as general diseases, antibiotic treatment 6 weeks before participation, alcohol consumption, smoking or pregnancy were also excluded [3, 9, 12, 24, 31, 49, 52, 55]. The selected sample size was set to 12 participants with specimens for each analysis in duplicates, which corresponds to studies with a similar study design and allows for the detection of large effect sizes >0.4 [4, 5, 27, 44, 61]. As the clinically observed lingual and buccal WSL formation upon orthodontic treatment differs by more than 60% [59], this experimental setup was considered sufficient. For biofilm collection, individual occlusal splints of the upper jaw were equipped with test specimens. The test specimens were placed buccally and palatally in the premolar and molar region in the first and second quadrants. This ensured an increased wearing comfort compared to the lower jaw and no impairment of aesthetics and phonetics for this initial study, but needs to be taken into account when evaluating the data. To avoid additional oral position-specific effects [4, 5], specimens were block-wise randomly assigned to the different evaluation groups. Instead of real brackets with geometries varying between different companies and especially between lingual and buccal appliances, uniform cylindric shaped bracket material was used. This ensures a higher reproducibility and comparability between the different positions. The test specimens were also not directly fixed to the splints, but to discs made of pure hydroxyapatite. This mineral is the main component of human enamel [15, 50] and the discs, thus, could be considered biomimetic alloplastic. The advantages of this construction are that it provided a standardized and uniform shape according to the desired dimensions, allowed for the analysis of the bracket/tooth interface, but avoided the use of allogeneic [35, 64, 67, 70] or xenogeneic material [2, 25, 33, 62]. This reduced ethical concerns regarding the study and increased participants acceptance. The decision not to use shielding in the area of biofilm collection—as was done in previous studies [16, 44, 63]—was based on the idea of simulating clinical reality as precisely as possible, i.e., allowing shear forces of the tongue and cheeks. Taken together, this splint design ensured almost natural biofilm formation that was close to the clinical orthodontic situation and, thus, can serve as the basis for further studies.

The splints were worn for 48 h by the participants. This time was set as other studies have demonstrated that it was appropriate for in situ biofilm growth [4, 5, 27, 35, 44, 51, 61, 64]. Due to the fact that oral hygiene was suspended, accumulation of biofilm formation was increased but it still should be considered as an initial biofilm. Even if the study design was not directly comparable with multiyear orthodontic treatment, it was sufficient for an initial study. However, it would be important for future studies to analyze the obtaining findings over the entire period of orthodontic treatment.

For the final analysis, half of the specimens were live/dead fluorescent stained and CLSM was used to quantify biofilm volume and live/dead distribution at the bracket/hydroxyapatite disc interface. This method is well established and allows for biofilm quantification with almost native morphology [17, 22, 29, 45, 60].

The detected biofilm consisted of bacterial and also human cells. These cells were most likely human gingival epithelial cells that are integrated into the biofilm through bacterial colonization, as has been demonstrated in various studies [16, 44, 63].

Compared to other studies, the biofilm volume was low in this study [16, 44]. This is most probably due to a different splint design in which the specimens were exposed to the natural conditions of the oral cavity, including shear forces by cheek and tongue, and were not protected by shield-like structures. However, the live/dead distribution was almost similar [44, 62].

When comparing the biofilms formed buccally and palatally, interestingly no statistically significant differences in volume or the live/dead distribution in this initial intraoral biofilm could be detected. This is in contrast to the study of Auschill et al. [5], which revealed a lower palatal biofilm thickness compared to the examined buccal locations. However, in their study the specimens were differently positioned buccally and palatally. Unlike the buccal specimens, the palatal specimens were placed in the area of the palatal mucosa at a greater distance from the teeth and the dental plaque. Other studies, which did not include palatal or lingual specimens, have shown similar vitality patterns at different locations on the buccal side of the upper and the lower jaw [4]. Taken together, there might be differences in biofilm formation capability at different locations in the oral cavity. However, for bracket-like specimens placed buccally and palatally in the upper jaw, the biofilm formed within the initial period seemed to be similar regarding its general morphology.

To gain deeper insight into acidification properties of biofilms, an analysis of the biofilm pH was carried out with a pH-sensitive ratiometric dye, followed by CLSM and digital image analysis software based on the protocol by Schlafer and Dige [57]. This quantitative fluorescence microscopic examination enables the determination of vertical and horizontal pH gradients in microscopic images without changing the biofilm mechanically, for example, with microelectrodes [57]. The pH was analyzed in different layers of the biofilm, due to the fact that the distribution of nutrients and metabolites in the biofilm is not even [57]. The heterogeneous biofilm has diffusion-modifying properties that cause chemical/nutrient gradients, which lead to microenvironments within the biofilm. Thereby, niches with different pathogenic potentials, such as pH, redox, and nutrient availability can develop [10, 23].

In line with this, in both oral positions the lowest pH value could be detected at the bottom layer. Buccally as well as palatally, the pH dropped likewise from 7.0 to approximately 6.45. The exact value strongly depends on the incubation conditions and the biofilm maturation state. The biofilm in this study was comparable young and incubated only for 45 min and with 0.4% glucose as sole nutrient source. This is most probably the reason for the likewise small drop in pH. An increase in specimen exposure time in the oral cavity, incubation time in glucose solution or additional nutrients available would most probably result in different outcomes. An increased biofilm volume and metabolism would lead to an increase in acid metabolic products that in turn would cause lower pH values, reaching the critical pH value for tooth enamel demineralization [1, 56]. Even though the biofilms in both oral positions showed similar pH values at the bottom layer, statistically significant differences could be observed between the pH values in the top layers 3 and 4. The biofilm grown on palatally exposed specimens showed a lower pH value than that grown buccally, which indicates stronger acidification by the bacteria present. With regard to the aforementioned results, this could not be due to greater palatal biofilm volume or higher viability. Most probably, the buffering effect of saliva from the parotid gland was responsible for the lower acidification buccally [42]. The excretory duct of the parotid gland enters the oral cavity opposite the second upper molar [38] and, thus, in closer proximity to the buccally placed specimens than to the palatal ones. A repetition of this study in the lower jaw could confirm the influence of salivary flow due to the different anatomical conditions. The excretory ducts of the sublingual gland are located close to the incisors and could potentially create a buffering effect that could lead to lower pH values buccally compared to lingually. Other studies have shown that WSL more frequently occurred in the maxillary than in the mandible arch [32, 34]. Gorelick et al. observed that WSL did not occur even after prolonged use of mandibular canine-to-canine bonded retainer and suggested salivary flow as a possible cause [26].

Another reason to consider for the lower acidification detected on the buccal side could be a different bacterial composition between the biofilms of the two oral positions. A consequence of different bacterial composition could be different metabolic activities and products and, thus, to differences in acidification [56]. To address this in more detail, future approaches should quantify bacterial metabolic activity buccally and palatally and identify specific species composition by genomic sequencing.

Regarding the initial hypothesis, it could be shown that the initial oral biofilms form similarly on bracket-like specimens if exposed buccally or palatally in the upper jaw. At both oral positions, the lowest pH value could be detected at the bottom layer of the biofilm, accordingly at the bracket/tooth interface. However, differences were determined at higher levels of the biofilm, which might be due to the specific position in the oral cavity. Further studies should address whether these observations can be confirmed also in the lower jaw with different anatomic properties and, most importantly, over longer periods of time close to the duration of orthodontic treatment. Changing the duration of the specimens in the oral cavity would most likely also result in different, lower pH values that would reach the critical pH for enamel demineralization [1, 56]. Another possibility to study especially the biofilm pH-caused demineralization in more detail would be to use the splint design of this study with bovine enamel instead of hydroxyapatite discs. Here, demineralization could be examined directly using digital transverse microradiography (TMR), a highly appropriate method for validating mineral losses [14]. Furthermore, previous etching of enamel, which is routinely done to increase surface energy, surface area, and porosity for efficient bracket adhesion [6, 11], could influence biofilm accumulation and should be considered in further studies. For example, Knösel et al. have shown that surplus etching of the entire labial surface results in a higher likelihood of developing WSL [37]. Therefore, the design and setup of this study could serve as a blueprint for multiple further analyses to examine pH development at the tooth–bracket interface in more detail.

Regarding site specific effects of demineralization, within the limitations of this study, there is no evidence for an intrinsic reduced biofilm formation capability at the palatal position. Therefore, further factors should also be considered for the clinically observed reduced WSL prevalence in lingual orthodontic treatment. For example, due to the custom-made manufacturing process [68], lingual brackets have a different geometry and the advantage of fitting precisely on the tooth surface [66]. In addition, they have a large base, which almost covers the entire lingual/palatal surface, thereby creating an inherent seal [66]. The specific effect of bracket geometry could be analyzed in further studies.

## Conclusion

In this study, a successful comparison between initial biofilm formation on palatally and buccally placed bracket-like material in the upper jaw with regard to acidification, biofilm volume, and live/dead distribution could be achieved. It could be shown that there were no differences in the general biofilm morphology regarding volume and viability. Interestingly, in biofilms from both positions a similarly decreased pH value at the deeper layers of the biofilm could be detected. Towards the top layers of the biofilms, the pH value increased at both positions. Only for palatally exposed specimens did the pH value slightly decrease from level 3 to level 4. Overall, a gradient from low pH values at the bottom layer to higher pH values at the top layer was discernible. Based on the results of this study, initial biofilm formation and acidification is similar on buccally and palatally placed bracket material in the upper jaw. As lingual brackets exhibit reduced WSL formation clinically, future studies should investigate further factors like bracket geometry.
